# AI is already in the theatre of war just not on the side of the patient: clinical chatbots, telemedicine, and an agenda for “AI for medical humanity”

**DOI:** 10.3389/fdgth.2026.1955671

**Published:** 2026-09-23

**Authors:** Andrew R. Prigg, Henry Robinson, Munzer Alkhalil, James Gough

**Affiliations:** 1 School of Medicine and Population Health, Sheffield Medical school, Sheffield, United Kingdom; 2 David Nott Foundation, London, United Kingdom; 3 Syria Public Health Network, London, United Kingdom

**Keywords:** artificial intelligence, chatbot, clinical decision support, humanitarian medicine, large language model

## Abstract

Artificial intelligence (AI) is already embedded in modern warfare, mainly through surveillance, targeting, and military decision-making. However, its potential to support clinicians and patients in conflict settings remains underdeveloped. Frontline medical teams must make time-critical, high-stakes decisions with scarce diagnostics, disrupted referrals, insecure communications, changing injury patterns, and irregular access to specialist expertise. Humanitarian medicine needs a proactive clinician-facing AI agenda that strengthens clinical judgment, not replaces it. We propose a two-stage model: a bounded, protocol-anchored chatbot as the “first mile” of point-of-care support, and telemedicine as the “second mile” for specialist oversight and accountability. The chatbot would structure assessments, guide protocol-based actions, identify missing information and areas of uncertainty, generate standard handovers, and trigger escalations. Remote specialists would provide context and retain responsibility for clinical decisions. Safe deployment requires transparent protocols, constrained scope, hard refusals, awareness of uncertainty, data minimisation, offline-first functionality, adversarial threat modelling, and safeguards against coercive or dual-use applications. Evaluation should use locally informed simulation, silent deployment, prospective monitoring of deviations and harms, predefined safety thresholds, and independent humanitarian oversight before wider clinical use. AI will increasingly shape conflict medicine, with or without humanitarian engagement. The priority is to ensure design, governance, and evaluation are guided by patient safety, medical neutrality, local involvement, and clinical accountability, placing AI on the side of the patient, not the machinery of war.

## Introduction: artificial intelligence in war, but not on the patient's side

Artificial intelligence (AI) is now part of the architecture of modern conflict. Its most visible trajectory has been towards automation of force, surveillance and targeting (1, 2). Meanwhile, clinicians working in field hospitals and improvised theatres continue to practise medicine with fractured infrastructure, constrained supplies and fragile access to expert advice (3). The question for global health is no longer whether AI will influence conflict settings, but whether we will build AI that expands the space for humane care rather than tightening it.

Recent BMJ Global Health work has outlined the broad opportunities for AI in conflict-affected health systems while emphasising profound risks: cybersecurity threats, privacy breaches, bias, weak accountability, and the weaponisation of health data (4). We share this caution. However, the breadth of “AI in conflict zones” can also make it hard to propose a concrete, testable agenda. Here, we focus on a narrower, actionable priority: *clinician-facing chatbots* in warzones, designed as bounded clinical copilots and integrated into telemedicine workflows.

## Before the battlefield: AI in clinical practice today

In high-income health systems, AI is no longer a future promise but a present clinical tool. It is already matching specialist performance in parts of medical imaging, supporting earlier detection in population screening, and helping clinicians interpret complex scans and pathology at scale (5). Across hospitals, predictive models are increasingly used to flag deterioration and sepsis risk earlier than traditional observation alone, while oncology is seeing growing use of AI to support risk stratification, pathway prioritisation, and treatment selection (6, 7). Beyond the hospital, AI is expanding remote monitoring through wearables and home devices, enabling earlier intervention in chronic disease and post-operative recovery (8). At the research frontier, it is accelerating drug discovery and biomarker development, tightening the loop between laboratory insight and bedside decision (9). The direction of travel is clear: from single-task decision support towards integrated systems that shape end-to-end clinical workflows, raising sharper questions about safety, accountability, and where human judgement must remain decisive. Yet almost all of these advances have been developed and evaluated in well-resourced health systems. Whether similar clinician-facing AI can safely support decision-making in conflict settings remains largely unexplored.

## A vignette: decision-making under fire in northern Syria

The following vignette is based on the first-hand clinical experience of one of the authors during the conflict in northwest Syria. Details have been anonymised and modified where necessary to protect patient confidentiality.

During the war in northwest Syria, an orthopaedic trainee and the head of a small hospital in Idlib faced multiple life-threatening emergencies simultaneously. A woman in obstructed labour required an urgent caesarean section; a man presented with severe abdominal haemorrhage; and a child needed a traumatic leg amputation following a nearby airstrike. The hospital had no surgeon on site. A general surgeon was two hours away and might not reach the facility. A volunteer intensive care specialist was available only remotely, from the United States. Transfers to other hospitals were impossible.

The hospital had just two intensive care beds, both occupied: one by a young woman ventilated for ten days with minimal chance of survival, and another patient in better clinical condition. While decisions were being made, a further bombing struck a nearby village. A man in his sixties arrived with a brain haemorrhage, clinically stable but requiring ventilatory support, with further casualties expected. Based on prior experience, there was a credible risk that the hospital itself would be targeted.

Within minutes, urgent decisions had to be taken: who should undergo surgery first, who should receive intensive care, whether to withdraw life support, whether to evacuate the hospital, and whether to reduce staff presence to limit risk. In this setting, the greatest challenge was not just a lack of clinical knowledge but making irreversible decisions under extreme uncertainty, time pressure, risk, and isolation

The question is, can AI tools alleviate uncertainty in such cases?

## Why chatbots matter in war medicine

Conflict care is not simply “medicine with fewer resources”. It is medicine under conditions that systematically produce uncertainty: disrupted referral pathways, limited diagnostics, fractured supply chains, rotating and mixed-skill teams, attacks on healthcare, and rapidly evolving injury patterns (3, 10). In these conditions, the most punishing deficit is often the absence of timely, reliable clinical reasoning support (11).

Chatbots can help because communication and information structuring are major bottlenecks in emergency care: Clinicians must rapidly translate chaotic scenes into structured assessments and management plans. Protocols are complex and hard to navigate under stress, fatigue and fear. Remote specialists spend scarce time reconstructing cases and clarifying missing details. Additionally, documentation and handover fail when teams rotate, facilities move, or communications degrade.

A clinical chatbot **If** properly designed, can reduce decision-making latency by guiding clinicians through the airway, breathing, circulation, disability and exposure (ABCDE) assessment and protocol-linked pathways, drawing on established WHO–ICRC emergency-care (12) and ICRC war-surgery guidance (3). These could include: haemorrhage control, damage-control principles, airway rescue, burns and crush management, obstetric emergencies, paediatric dosing, and sepsis recognition. In warzones, however, a ‘chatbot’ cannot be a generic, internet-trained conversational agent. It must be a bounded clinical system with clearly defined functions and deliberate safety safeguards.

## Telemedicine and chatbots should function as one system

A recurring challenge for telemedicine in conflict and resource-constrained settings is unreliable connectivity. Limited bandwidth and high latency can make synchronous video calls brittle, with interruptions reported across multiple interventions, while audio-based approaches are often more resilient (13). Telemedicine has still been used to bridge specialist shortages. In Syria, for example, remote ICU ward rounds were supported using low-cost digital tools and predominantly WhatsApp communication with specialists abroad (14). Such models can work, but they are labour-intensive and fragile when bandwidth, time zones and security constraints collide (13, 15).

One way to reduce this dependence on a continuous signal is to pair telemedicine with chatbots as the “first mile” of care. A chatbot that can function with minimal data, or in an offline-first mode that stores inputs locally and uploads when connectivity returns, could structure histories, capture key observations, and surface triage red flags. This enables a safer store and forward workflow: remote specialists receive standardised case packets to review asynchronously, verify, nuance, and explicitly own decisions, rather than trying to make high stakes calls through unstable real-time links.

### A practical suggested operating model: first-mile chatbot, second-mile telemedicine

We propose a two-stage operating model integrating point-of-care chatbot support with specialist telemedicine oversight (Figure 1).

**Figure 1:**
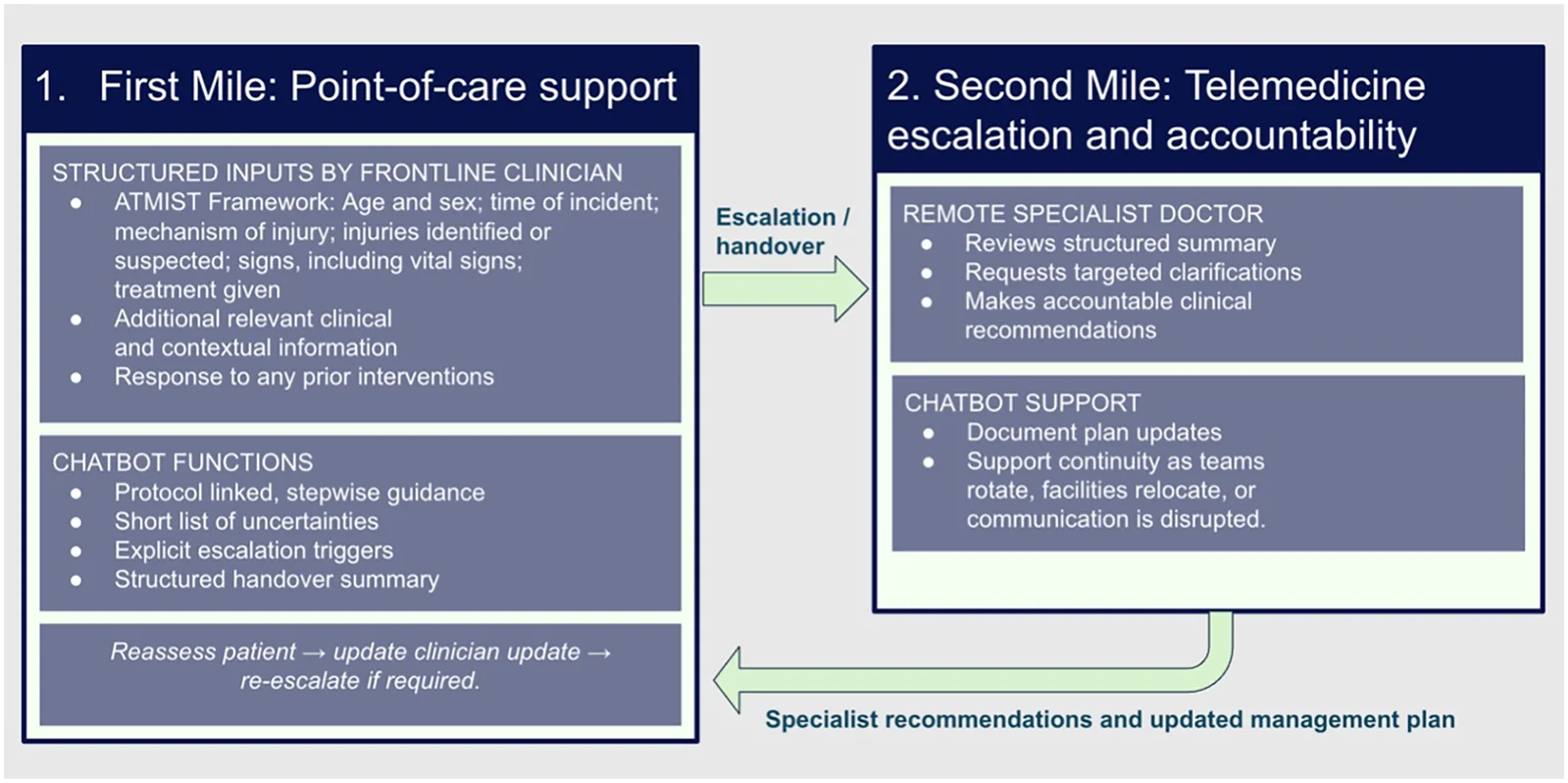
Proposed operational model integrating a first-mile clinical chatbot with second-mile telemedicine.

#### First mile (at the point of care)

Minimum structured inputs should follow the ATMIST framework: age and sex; time of incident; mechanism of injury; injuries identified or suspected; signs, including vital signs; and treatment given. These should be supplemented by additional relevant clinical and contextual information and the patient's response to any prior interventions (16). A well-designed chatbot should provide protocol-linked stepwise guidance; a short list of uncertainties; explicit escalation triggers; and a structured handover summary.

#### Second mile (telemedicine escalation and accountability)

Remote specialists would review the structured summary, request targeted clarifications, and make accountable clinical recommendations. The chatbot would document plan updates and support continuity as teams rotate, facilities relocate, or communication is disrupted.

## What “AI on the side of the patient” should require of warzone chatbots

Any chatbot intended for conflict care must be designed for hostile infrastructure and coercion risk concerns repeatedly raised in conflict-zone AI discussions (4). We suggest a small set of non-negotiable design requirements.

These requirements align with global guidance on AI ethics in health and on responsible humanitarian uses of AI (17).

### Non-negotiables

- Protocol anchoring and source transparency: recommendations should cite the approved protocol/guideline step they rely on, and surface assumptions and missing information.
- Bounded clinical knowledge base: the chatbot's protocol library should be restricted to a fixed, version-controlled corpus of up-to-date clinical content — for example, curricula such as the David Nott Foundation's Hostile Environment Surgical Training (HEST®) programme — with the system declining to respond and escalating to telemedicine when a question falls outside this scope (18). Restricting the knowledge domain in this way constrains the space in which hallucination can occur, rather than relying on the underlying language model's general training to produce dependable behaviour.
- Constrained scope with hard refusals: the system must refuse requests that support targeting, interrogation, profiling, coercive surveillance, or other dual-use functions that blur care and harm.
- Uncertainty-aware behaviour: when information is incomplete or risk is high, the default output should be to request clarifications and encourage escalation, not to improvise confidently.
- Data minimisation and offline-first assumptions: store as little identifiable data as possible, encrypt by default, and design for intermittent connectivity and power interruption.
- Adversarial threat modelling: treat devices and networks as contested; plan for confiscation, extraction attacks, re-identification and manipulation.

## How to evaluate chatbots safely in unstable environments

Scepticism is justified: fluency can be mistaken for correctness, hallucinations can be dangerous, and automation bias can erode clinician judgement. These risks intensify where redundancy and oversight are limited. However, healthcare is beginning to generate practical deployment models that move beyond hype. In peri-operative medicine, the PEri-operative AI CHatbot (PEACH) embedded institutional protocols into a secure environment. It evaluated performance using “silent deployment”: clinicians made decisions first, then compared chatbot outputs against guidelines and expert consensus. Across 240 real-world clinical iterations, accuracy was high and deviations/hallucinations were explicitly audited for potential harm, with iterative updates when deficiencies were detected (19).

Conflict settings involve weaker infrastructure and greater security and coercion risks, but the underlying evaluation logic remains applicable. This claim concerns evaluation methodology, not clinical performance: we are not assuming PEACH's clinical outcomes would replicate in a conflict setting, only that its staged, audited evaluation process is adaptable across resource and connectivity constraints. Evidence for this adaptability spans a widening gradient: generative AI-enabled clinical decision support evaluated by cluster-randomised trial in high-resource primary care (19, 20), mixed-methods evaluation of clinician adoption of similar tools in Kenya, alongside an ongoing multi-facility trial comparing large language model-assisted and unassisted clinicians in Nairobi (21). A feasibility study developing chatbot-enabled decision support explicitly for Delayed/Disconnected, Intermittently Connected, Low-Bandwidth conditions in military medicine (22), the closest existing technical analogue to conflict connectivity constraints.

Clinical performance generalisability itself remains untested and is the specific object of the evaluation pathway proposed below, not a premise of it.

Testing should begin with simulation and tabletop exercises involving local teams to assess prompts, edge cases, resource constraints, and potential failure modes. This should be followed by silent deployment within routine workflows, comparing chatbot outputs with agreed protocols and clinician decisions without influencing care. A prospective registry should document interactions, deviations, near-misses, and potential harms using a predefined severity classification. Clinical use should then expand gradually, and only after predefined safety thresholds have been met and an independent governance review has been completed.

Because these outcomes bear directly on patient safety, future evaluation should be assessed against predefined, measurable endpoints. These should include protocol-adherence rate, comparing chatbot output against a fixed gold-standard protocol answer; hallucination/critical-error rate per 100 interactions, audited during silent deployment; decision-to-handover latency; escalation-trigger sensitivity and specificity relative to clinician judgement; and connectivity failure and recovery rate under offline-first operation. Restricting the chatbot's knowledge base to a defined protocol corpus, as described above, is intended to reduce the incidence of the errors these metrics are designed to detect, rather than to substitute for their prospective measurement.

## Anticipated objections (and why they do not end the conversation)

Three concerns are likely to dominate discussions of warzone chatbots:

“**Chatbots will worsen automation bias.”** They might - unless designed to support reasoning rather than replace it. Protocol-cited outputs, uncertainty flags, and mandatory escalation triggers can keep clinicians in control, with telemedicine acting as the accountability layer.

“**These systems will be weaponised.”** Dual-use is real. The response is not to avoid clinical tools altogether, but to set hard scope limits, refusal behaviours, and humanitarian oversight explicitly linked to medical neutrality and international humanitarian law.

“**Data security is impossible in war.”** Perfect security is unrealistic; unmanaged risk is unacceptable. Data minimisation, offline-first design, encryption, and adversarial threat modelling reduce harm even when devices are seized or networks are monitored.

## Towards an agenda for “AI for medical humanity”

If AI in conflict zones is left entirely to technology companies and militaries, the gravitational pull will remain towards smarter weapons and more efficient killing. The global health and humanitarian community should instead articulate a proactive agenda for AI that widens the space for humane care, including:

- Participatory priority-setting with frontline clinicians, patients and communities in multiple conflict settings, to define which AI tools are genuinely needed.
- Development of evaluation frameworks suited to unstable environments combining simulation, prospective registries and pragmatic trials where feasible.
- Establishment of governance and ethics mechanisms including independent humanitarian oversight bodies to review proposed AI tools for conflict use, analogous to ethics review boards.
- Normative work linking AI governance to international humanitarian law and medical neutrality, including clear prohibitions on dual-use systems that blur the line between care and targeting.
- Targeted funding and capacity-building so that expertise in AI design and critique is not confined to high-income countries and military contractors.

## Conclusion

For clinicians reading this in relative safety, the idea of AI systems designed specifically for war medicine may feel uncomfortably futuristic. But AI is already in the theatre of war -just not usually on the side of the patient. The question is no longer whether AI will shape conflict medicine, but how, and under whose values.

If we do nothing, AI will continue to flow along its current channels: surveillance, targeting and profit. If, instead, we choose to invest intellectual, ethical and financial capital in tools that help clinicians in the most dangerous places on earth to practice safer, more compassionate medicine, then “*AI for medical humanity, not lethality*” can be more than a slogan. It can become a design principle - and, for our patients in war, a lifeline.

## Data Availability

The original contributions presented in the study are included in the article/Supplementary Material, further inquiries can be directed to the corresponding authors.
